# Clinical predictors for mechanical ventilation assistance in Guillain-Barré syndrome

**DOI:** 10.3389/fneur.2024.1385945

**Published:** 2024-05-09

**Authors:** Axel Abel Rodríguez-Méndez, Jaime Briseño-Ramírez, Francisco Javier Rivas-Ruvalcaba, Javier Solis-Estrada, Liliana Berenice Alcázar-García, Karely Díaz-Ramírez, Gabriela Lira-Jaime, Edgar Javier Sánchez-Román, Carlos Zúñiga-Ramírez

**Affiliations:** ^1^Department of Internal Medicine, Hospital Civil de Guadalajara “Fray Antonio Alcalde”, Guadalajara, Mexico; ^2^Health Division, Tlajomulco University Center, University of Guadalajara, Guadalajara, Mexico; ^3^Department of Neurology, Hospital Civil de Guadalajara “Fray Antonio Alcalde”, Guadalajara, Mexico

**Keywords:** Guillain-Barré syndrome, mechanical ventilation, respiratory failure, acute autoimmune polyneuropathy, clinical predictor

## Abstract

**Background:**

Guillain-Barré syndrome (GBS) frequently leads to respiratory failure and autonomic dysfunction, resulting in approximately one-third of patients requiring mechanical ventilation.

**Objective:**

This study aimed to identify clinical predictors for mechanical ventilation in patients with GBS.

**Methods:**

This research was conducted from 2010 to 2021 using registries from a tertiary hospital in an upper middle-income Latin American country. Participants were categorized into two groups based on their ventilation status. Demographic data were collected, and independent predictors of the need for mechanical ventilation were determined through multivariate logistic regression analysis.

**Results:**

Dysautonomic events occurred in 36% of the patients, with 17% requiring mechanical ventilation; the average duration of intubation was 1.16 ± 3.18 days. The multivariate analysis indicated that bulbar dysfunction significantly increased the likelihood of requiring mechanical ventilation by 19-fold (OR 18.67, 95% CI 5.85–59.42), followed by ophthalmoplegia, which increased the likelihood by sixfold (OR 5.68, 95% CI 1.28–25.19).

**Conclusion:**

Bulbar dysfunction, dysautonomia, and lower Medical Research Council (MRC) scores were significant predictors of the need for mechanical ventilation in hospitalized GBS patients. These findings support the need for close monitoring and early admission to the intensive care unit (ICU) admission for at-risk patients.

## Introduction

Guillain-Barré syndrome (GBS) is an acute immune-mediated demyelinating polyradiculoneuropathy. It was first identified over a century ago. Despite medical advancements, it remains a leading cause of acute non-traumatic flaccid paralysis (1–4). The global incidence rates of GBS are estimated at 1–2 cases per 100,000 person-years, and these rates increase with age and exhibit a male predominance (5). Clinically, GBS presents as an acute, progressive, symmetric bilateral weakness with hyporeflexia or areflexia. Typically, the condition reaches its nadir approximately 4 weeks after the onset of symptoms (3, 4). Respiratory failure and autonomic dysfunction are life-threatening complications seen in the advanced phase of the condition. The mortality rate varies from 1 to 18% for those requiring mechanical ventilation (6–8).

Frequently, a gastrointestinal (GI) or respiratory infectious event occurs 4 weeks before the neurological manifestations of Guillain-Barré syndrome (GBS) become apparent (1, 3, 4). There is substantial evidence supporting the interaction between *Campylobacter jejuni* infection and the development of GBS through molecular mimicry (3). However, other infectious agents from the GI and respiratory tracts are also associated with this condition (4, 5). In Latin America, 30–65% of GBS cases are classified as acute motor axonal neuropathy (AMAN) and acute motor and sensory axonal neuropathy (AMSAN) subtypes. Both subtypes, AMAN and AMSAN, are often preceded by *C. jejuni* infection and tend to progress to a severe clinical course. Conversely, in other regions such as North America and Europe, acute inflammatory demyelinating polyneuropathy (AIDP) is the most common form of the disease, with the AMAN variant reported in only 10% of cases (9).

Bragazzi et al. (10) discovered that Mexico, classified as an upper middle-income country, exhibits a higher prevalence of Guillain-Barré syndrome (GBS) compared to other nations. They also observed an increase in its incidence from 1990 to 2019 along with a greater-than-expected disease burden, as measured by years lived with disability. Furthermore, management, outcomes, and costs associated with GBS vary significantly between high-income and low-income countries (11). For example, van Wagenberg et al. (12) reported that, in Ethiopia, a low-income country, GBS-related treatment expenses exceeded USD$ 68 million, with an average cost of USD$ 267 per case. A study from the Netherlands found that the cost of treatment increased with the severity of the disease, ranging from 2,428 euros for patients with a Hughes score of 1 to 59,167 euros for those with a score of 5 (13).

Up to one-third of all GBS cases require invasive mechanical ventilation (IMV) due to acute respiratory failure. Among these cases, 10–20% result in fatality because of pneumonia, sepsis, or respiratory arrest (7, 14–21). Previous studies have identified risk factors that predict the need for IMV, such as older age, male sex, cranial nerve deficit, lower MRC score, shorter interval between neurological symptoms and hospital admission, dysautonomia, and axonal variants, among others (22–24). Interestingly, severe median nerve damage has also been identified as an independent factor for prolonged IMV (25). However, early clinical predictors for IMV have not been sufficiently described in populations where variants different from AIDP are more common. This study aimed to identify clinical predictors of IMV among patients with initial features of GBS.

## Materials and methods

### Ethics statement

This study was approved by the local ethics committee and performed according to the Helsinki declaration. Since this study was performed retrospectively, no informed consent from the studied subjects was needed.

### Population and eligibility criteria

We reviewed the medical records of patients admitted to the hospital with the following International Classification of Diseases 10th Edition (ICD-10) codes: (1) G122: neuron-motor disease, unspecified (2) G610: Guillain-Barré Syndrome (3) G618: other inflammatory demyelinating polyneuritis, and (4) G628: other specified polyneuropathies. These records were obtained from patients admitted during 2010–2021.

For GBS, we included patients who met either Brighton or National Institute for Neurological Diseases and Stroke (NINDS) criteria, as well as the Brighton Collaboration Group criteria for Miller-Fisher syndrome, provided they were older than 15 years of age (26).

To classify neurophysiological variants, the electrophysiological diagnostic criteria by Hadden were used (27). Patients younger than 15 years of age and with more than 10% of missing data from clinical records were excluded. Demographic data, the presence of precipitating factors, clinical variant subtype of GBS, and other clinical data were collected from medical records. This included examination findings such as vital signs at admission, initial laboratory studies, the pattern of progression of weakness, the MRC weakness scale, the Hughes disability scale upon admission, the presence of bulbar and other cranial nerve dysfunctions, and the use of IMV. Additionally, we identified the presence of dysautonomia, defined in the clinical records as either hyperactivity or autonomic failure, characterized by fluctuations in blood pressure, cardiac arrhythmias, ileus, or urinary retention.

We calculated the sample size using the formula of Marragat et al. (28) to estimate the differences between independent proportions, using the proportion of GBS cases requiring mechanical ventilation described by Fourrier et al. (29). Assuming a power of 80% and an alpha value of 5%, we estimated that for every three cases not requiring IMV, there would be one case requiring IMV. In total, 33 subjects requiring IMV and 99 not requiring IMV were needed to detect a statistically significant difference between the groups. Additionally, we estimated missing data of 5%.

### Statistical analysis

Demographic data were reported as simple relative frequencies. The normality of data distribution was assessed using the Shapiro–Wilk test. Pearson’s chi-squared and Fisher’s exact test were used to compare proportions. For comparison of quantitative variables, Student’s T and Wilcoxon-Mann–Whitney tests were used for normal and non-normal distributions, respectively.

A multivariate model was used through a logistic regression to identify independent factors related to IMV. Variables included in the model had a *p*-value of <0.1 and were biologically plausible. A step-backward selection of the variables for the model was performed. The Hosmer-Lemeshow test was used to determine the goodness of fit, with values greater than 0.1 considered appropriate. The confidence intervals were defined as 95% (CI = 95%). STATA package version 17 was used for statistical analysis (College Station, TX, USA).

## Results

**Figure 1:**
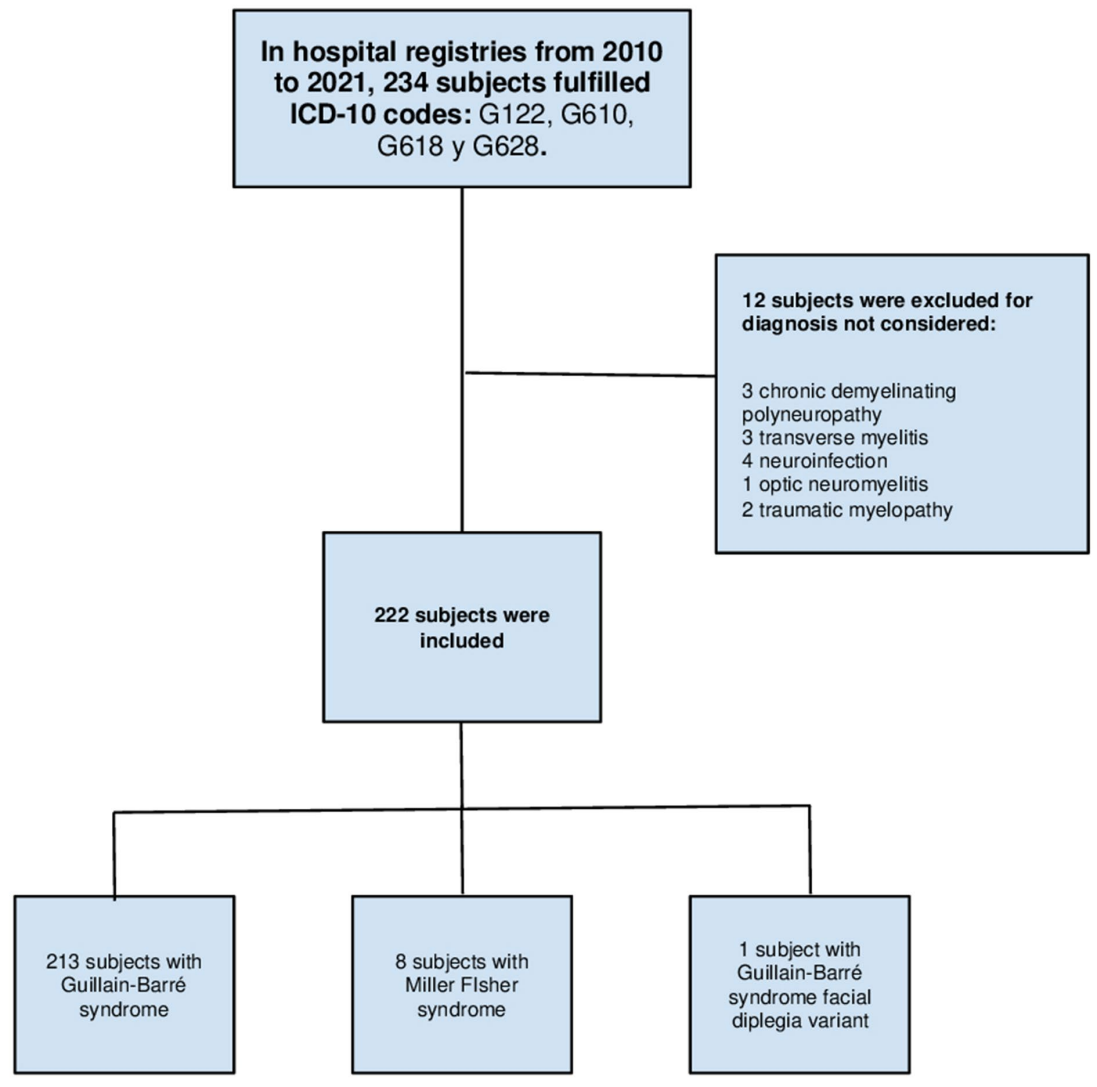
Flow chart of clinical records search. ICD, International Classification of Diseases.

From the search in hospital registries, 234 subjects were collected, out of which 222 were included based on the selection criteria, as depicted in Figure 1. Of the cases included, the mean age was 39.7 ± 16.51 years (median 40, ranges from 15 to 79 years), 60.8% were male (*n* = 135), and 95.5% presented the classic clinical variant of GBS (*n* = 213). The remaining sociodemographic data and baseline characteristics of the subjects are found in Table 1.

**Table 1:** Sociodemographic and baseline characteristics of patients with Guillain-Barré syndrome.

Characteristics	Total	Men	Women	p*
N (%)	222 (100)	135 (60.81)	87 (39.19)	0.001
Mean age (SD)	39.7 (16.51)	37.46 (15.66)	43.2 (17.28)	0.016
Comorbidities - N (%)				
Diabetes mellitus	25 (11.26)	11 (44)	14 (56)	0.055
Hypertension	33 (14.86)	14 (42.42)	19 (57.58)	0.022
Active smoking	28 (12.61)	23 (82.14)	5 (17.86)	0.013
Alcohol consumption	34 (15.32)	30 (88.24)	4 (11.76)	<0.001
Clinical variant - N (%)				0.55
Classic form	213 (95.95)	130 (61.03)	83 (38.97)	
Miller Fisher	8 (3.6)	5 (62.5)	3 (37.5)	
Facial diplegia	1 (0.45)	0 (0)	1 (100)	
Infectious precedent - N (%)				
Gastrointestinal	79 (35.59)	48 (60.76)	31 (39.24)	0.99
Respiratory tract	55 (24.77)	31 (56.36)	24 (43.64)	0.43
Current immunization* - N (%)	14 (6.31)	6 (42.86)	8 (57.14)	0.17

*Influenza and/or SARS-CoV-2 immunization.

Based on the studies of nerve conduction and electromyography for the classification of the electrophysiological variant of the subjects with GBS, 171 observations were collected, the remaining were classified as indeterminate due to non-specific findings in the study that failed to discern the variant at the time of its performance (such as persistent F waves as the only finding) or for missing neuroconduction study data. The AMAN variant was the most frequent with 46.4% of the cases (*n* = 103), followed by the AMSAN variant in 19.37% of the observed patients (*n* = 43), and the AIDP variant was reported in 9.9% of the cases (*n* = 22).

It was found that 68.47% of observed cases had a pattern of weakness progression that increased (*n* = 152) when the neurophysiological variants were associated with the clinical characteristics. The mean hospital admission time since symptom onset was 5.61 ± 4.3 days (median 4, ranging from 1 to 30 days), and the mean for the nadir time to weakness was 4.6 ± 3.43 days (median 3, ranging from 1 to 21 days). The degree of muscle strength and stretch reflexes in various muscle groups of the upper and lower extremities were evaluated. The global scale of the MRC had a mean score of 30.2 ± 16.05 points (median 30, ranging from 0 to 60 points). Hyporeflexia in the upper limbs was the most common feature, manifesting in 59% of the subjects (*n* = 131).

Bulbar dysfunction was the most common manifestation of cranial neuropathies in 29% of the patients (*n* = 65). Using the disability scale in patients with GBS, proposed by Hughes et al. (30), 52.46% of the cases were bedridden (*n* = 64). Furthermore, 36% of the patients had dysautonomia (*n* = 81): extreme fluctuations in blood pressure, arrhythmias, ileus, and/or urinary retention. The full set of clinical variables within neurophysiological subtypes is depicted in the Supplementary Tables 1, 2.

Concerning the need for IMV, 17.12% of the cases required this intervention (*n* = 38), and the average intubation time from the onset of symptoms was 1.16 ± 3.18 days. Of these cases, 10% died during their hospitalization (*n* = 4), and only 54% received care in the intensive care unit (*n* = 20). For those cases that required invasive ventilatory support, the mean age was 38.97 ± 17.05 years (median 38.5, range from 15 to 76 years), and 60.52% of the cases were men (*n* = 23). Regarding the clinical subtype, 97.36% of the patients requiring IMV were classified as the classic form (*n* = 37), and only 1 subject had Miller-Fisher syndrome. Concerning the neurophysiological variant and need for IMV, 31.37% of the indeterminate variant cases required IMV (*n* = 16) compared with 12.62% of the patients with the AMAN variant (*n* = 13) and 18.6% (*n* = 8) of the AMSAN variant cases. Finally, only 4.55% of the cases that required mechanical ventilation were related to the AIDP variant (*n* = 1).

In the univariate analyses, the presence of bulbar dysfunction and dysautonomia showed a positive association with the requirement for IMV. Similarly, the presence of gastrointestinal infection as a trigger for GBS demonstrated a negative association with the requirement for IMV. Significant differences were found in heart rate, respiratory rate, SpO_2_, MRC weakness scale, lymphocyte count, and systemic inflammatory index among subjects who required IMV compared to those who did not. The remaining comparisons are shown in Table 2.

**Table 2:** Baseline, clinical, and biochemical features regarding invasive mechanical ventilation requirement.

Variable	Obs.*	IMV requirement	Not IMV requirement	*p*
N (%)	222	38 (17.12)	184 (82.88)	**<0.001**
Mean age (SD)	222	38.97 (17.05)	39.86 (16.45)	0.78
Women - N (%)	222	15 (17.24)	72 (82.76)	0.97
Clinical variant - N (%)	222			
Classic form		37 (17.37)	176 (82.3)	0.99
Miller-Fisher		1 (12.5)	7 (87.5)	
Facial diplegia		0 (0)	1 (100)	
Neurophysiological variant N (%)	222			
Indeterminate		16 (31.37)	35 (68.63)	**0.026**
AMAN		13 (12.62)	90 (87.38)	
AMSAN		8 (18.6)	35 (81.4)	
AIDP		1 (4.55)	21 (95.45)	
ASAN		0 (0)	3 (100)	
Comorbidities - N (%)	222			
Diabetes mellitus		5 (20)	20 (80)	0.77
Hypertension		4 (12.12)	29 (87.88)	0.61
Active smoking		4 (14.29)	24 (85.71)	0.79
Alcohol consumption		7 (20.59)	27 (79.41)	0.56
Precedents - N (%)	222			
GI infection		12 (15.19)	67 (84.81)	0.57
Respiratory tract infection		12 (21.82)	43 (78.18)	0.29
Immunization		5 (35.71)	9 (64.29)	0.07
MRC weakness scale - μ (SD)	222			
Global weakness		15.21 (12.85)	33.38 (14.85)	**<0.001**
Upper limbs weakness		8.42 (7.39)	18.20 (8.16)	**<0.001**
Lower limbs weakness		6.78 (6.3)	15.21 (8.47)	**<0.001**
Dysautonomia - N (%)	222	29 (35.8)	52 (64.2)	**<0.001**
Weakness progression pattern - N (%)	222			
Ascendent		27 (17.76)	125 (82.24)	0.7
Descendent		11 (15.71)	59 (84.29)	
Upper limbs myotatic reflexes - N (%)	222			
Arreflexia		19 (31.67)	41 (68.33)	**0.003**
Hyporreflexia		17 (12.98)	114 (87.02)	
Normorreflexia		1 (3.85)	25 (96.15)	
Hyperrreflexia		1 (20)	4 (80)	
Lower limbs myotatic reflexes - N (%)	222			
Arreflexia		25 (26.32)	70 (73.68)	**0.021**
Hyporreflexia		12 (10.71)	100 (89.29)	
Normorreflexia		1 (8.33)	11 (91.67)	
Hyperreflexia		0 (0)	3 (100)	
Cranial neuropathy - N (%)				
Facial palsy	222	5 (13.89)	31 (86.11)	0.8
Bulbar dysfunction	222	31 (47.69)	34 (52.31)	**<0.001**
Ophtalmoplegia	222	8 (25.81)	23 (74.19)	0.17
Vital signs - μ (SD)				
SpO2	221	92.84 (4.7)	95.36 (3.47)	**<0.001**
RR	221	21.46 (4.87)	17.99 (2.8)	**<0.001**
HR	222	93.39 (20.24)	81.61 (12.73)	**<0.001**
Biochemical - μ (SD)				
Hemoglobin	220	15.33 (2.23)	15.44 (1.98)	0.96
Platelets	220	270.24 (99.27)	282.05 (87.18)	0.36
Leukocytes	220	12.72 (6.03)	10.54 (4.37)	0.1
Lymphocytes	220	1.83 (0.6)	2.43 (1.21)	**0.001**
SII	220	1765.89 (1437.21)	1061.75 (907.6)	**0.003**
Glucose	218	112.71 (42.4)	105.84 (40.5)	0.17
eGFR	221	108.1 (27.58)	113.87 (21.96)	0.29
Na+	215	137.51 (5.59)	136.38 (4.05)	0.54
K+	215	4.02 (0.57)	3.98 (0.46)	0.79
Ca++	210	9.32 (0.84)	9.32 (0.67)	0.48
Mg++	41	1.92 (0.47)	2.11 (0.21)	0.46
LDH	179	185.79 (72.96)	179.66 (69.52)	0.36
AST	190	40.9 (44.17)	44.74 (60.2)	0.47
ALT	190	39.97 (39)	49.81 (86.74)	0.65
Albumin	189	3.92 (0.59)	3.92 (0.61)	0.98
CK	111	197.17 (191.55)	218.85 (384.89)	0.86

IMV, invasive mechanical ventilation; AMAN, acute motor axonal neuropathy; AMSAN, acute motor and sensory axonal neuropathy; ASAN, acute sensory axonal neuropathy; AIDP, acute inflammatory dyemilinating polyneurpathy; GI, gastrointestinal; MRC, Medical Research Council; SO_2_, oxygen saturation; RR, respiratory rate; HR, heart rate; SII, systemic inflammatory index (SII = P × N/L, where P, N, and L are peripheral platelet, neutrophil, and lymphocyte counts, respectively); eGFR, estimated glomerular filtration rate; LDH, lactate dehydrogenase; ALT, alanine aminotransferase; AST, aspartate aminotransferase; CK, creatine kinase.

*Observations frequency.

Bold values are for statistically significant variables.

**Figure 2:**
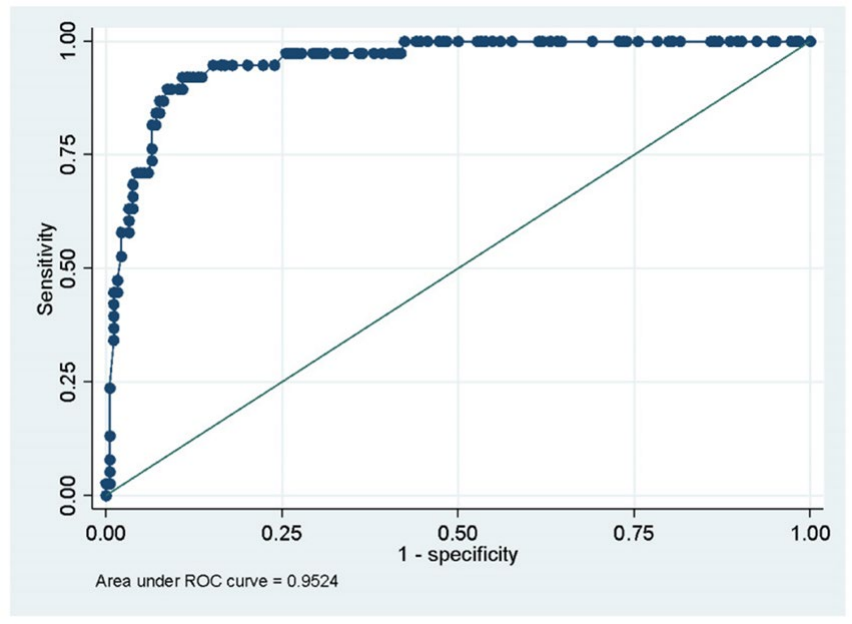
The area under the curve ROC.

A logistic regression analysis was performed, with a step-backward procedure for significant independent variables, considering a *p*-value of <0.1 for its adequacy. Five variables were of statistical significance, resulting in two variables within the group of cranial neuropathies. First, bulbar dysfunction significantly increased the odds, almost 19 to 1, of requiring IMV (OR 18.67, 95% CI 5.85–59.42), followed by the presence of dysautonomia (OR 11.55, 95% CI 3.35–39.89) and ophthalmoplegia (OR 5.68, 95% CI 1.28–25.19). A higher MRC score during admission correlated with the diminished chance of requiring IMV (OR 0.91, 95% CI 0.88–0.95). The same is true regarding gastrointestinal infection as a trigger of GBS (OR 0.26, 95% CI 0.08–0.9; Table 3). Goodness of fit was adequate for this model (Hosmer-Lemeshow test, *p* = 0.91). This model also showed a high discriminative capacity as seen in the ROC curve (AUC 0.95; Figure 2).

**Table 3:** Multivariate analysis regarding mechanical ventilation requirement.

Variable	OR	CI 95%	*p*
Dysautonomia	11.55	3.34–39.89	<0.001
Bulbar dysfunction	18.67	5.85–59.42	<0.001
MRC scale of global weakness	0.91	0.87–0.95	<0.001
Ophthalmoplegia	5.68	1.28–25.19	0.022
GI infection	0.25	0.07–0.89	0.003

MRC, Medical Research Council; GI, gastrointestinal.

Hosmer Lemeshow value *p* = 0.91.

Pseudo *R*^2^ = 0.52.

In terms of medical treatment and therapy, 215 cases received immunomodulatory therapy, with plasmapheresis being the most common therapy given to 53.6% of the patients (*n* = 119), followed by immunoglobulin provided to 43.24% of the cases (*n* = 96). The average time from therapy administration to the symptom onset was 7.32 ± 4.37 days (median 7, range 0–28 days). During their hospital stay, only 88 patients (39.64% of the total) were evaluated by a neurologist, of which 25 were on assisted mechanical ventilation (11% of the total cases). The in-hospital mortality rate was 1.8% (*n* = 4).

## Discussion

In our analysis, we found that the main variables associated with an increased risk of respiratory failure and the need for invasive mechanical ventilation in patients with Guillain-Barré syndrome were the presence of bulbar dysfunction, ophthalmoplegia, dysautonomia, and a lower Medical Research Council (MRC) score. Conversely, it is suggested that the onset of the disease following a gastrointestinal event may decrease the risk of requiring IMV.

Our study contributes to the limited knowledge concerning risk factors for clinical features associated with the need for ventilatory support in middle-income countries. Currently, only two studies addressing this issue are available in the literature: a narrative review conducted in Mexico in 2013, and a retrospective cohort study carried out in Peru in 2021. Otherwise, the studies that have been published were conducted in high-income countries, specifically in Europe and the United States (32, 33–39).

From the data we obtained, the presence of bulbar dysfunction is the feature most strongly associated with the need for IMV in GBS, with an odds ratio of 18.67 (95% CI 5.85–59.42). This finding suggests that bulbar dysfunction may serve as an indicator of the progression of respiratory muscle weakness, as well as a condition for poor secretion management, and inadequate airway protection. This observation is consistent with a recent study conducted in Bangladesh (32), where the clinical characteristics of the cases were similar to our study, including the predominance of the AMAN variant. Additionally, the Erasmus GBS Respiratory Insufficiency Score (EGRIS) proposed by Walgaard et al. (35) is a tool used to identify patients who are likely to require IMV within the first 7 days. According to EGRIS, bulbar dysfunction, along with facial paralysis and a low Medical Research Council (MRC) score, was found to be a significant predictor.

In a 2017 study conducted in Thailand, the combination of bulbar dysfunction and rapid development of severe weakness were identified as risk factors associated with the need for mechanical ventilation in patients with GBS; however, this finding was in a subset of patients who were ventilated for more than 15 days (16). In contrast, a retrospective cohort study from Mexico carried out by Malaga et al. (34) did not report a statistically significant association between bulbar dysfunction and the group of ventilated GBS patients (30% of them with IMV), nor between severe weakness and a low Medical Research Council (MRC) score (70% of them with IMV). The authors suggested that this may be attributed to the low availability of ventilators and inconsistencies in the criteria for selecting cases for IMV.

In our study, the presence of dysautonomia increased the probability of developing ventilatory failure and necessitating IMV, with an odds ratio (OR) of 11.5 (95% CI 3.34–39.89). This factor was similarly identified by Wen et al. (31), who observed that dysautonomia events had an odds ratio of 6.42 (95% CI 619.58–17,111.89) for requiring IMV, coupled with a low Medical Research Council (MRC) score at nadir (OR –0.13, 95% CI 0.79–0.98). These findings concur with our research, which demonstrates an association between a lower MRC score and a higher probability of requiring IMV (OR 0.91, 95% CI 0.87–0.95). This last variable was identified as an independent risk factor for the need for invasive ventilatory support in studies by Islam et al. (32) and another study of the North American population conducted by Lawn et al. (30). Nonetheless, given the significance of dysautonomia, it is necessary to establish clinical protocols to identify this condition before the development of ventilatory failure.

Interestingly, our analysis suggests that patients with GBS with a history of gastrointestinal infection had a lower probability of requiring IMV. A strong association between the AMAN variant presentation and gastrointestinal infection by *C. jejuni* is known, and it has been hypothesized that, despite being a more severe presentation variant, it generally spares the proximal muscles and cranial nerves (35). This may explain its association with reduced odds for IMV necessity. While another hypothesis proposes that the demyelinating GBS variants are related to a greater development of respiratory failure (36), in our study, only one patient with the AIDP variant required invasive ventilatory support.

A clinical trial performed in Paris from 2004 to 2005 (37) evaluated the outcomes of patients with GBS who developed respiratory failure, comparing a group with early IMV against another with non-invasive management. The authors did not find any significant differences between these groups in the development of pneumonia associated with ventilation, distressed respiratory, or death. Nonetheless, the inherent disparities between European population, the availability of resources, and the specialized attention of an ICU in contrast with a general ward from a low-income country makes this analogy unreasonable.

The limitations of our study are mainly due to its retrospective nature. First, while collecting data, some variables were missing, which had to be excluded from the analyses. Moreover, some variables that were intended for inclusion were not found in the search within the hospital records. Second, due to the retrospective nature of the study, biochemical tests were only obtained at admission. Although some of these tests (such as the lymphocyte count and the systemic inflammatory index) showed significant differences in the outcome, they represent only initial observations and thus were not included in the multivariate model.

To ensure accurate and reliable results, it is recommended to conduct additional studies on the same population and region using prospective cohorts or during the development of clinical trials. This will provide the opportunity to include more data and better control variables to be included. It should be noted that the information obtained in this study is limited to a single hospital center, which may hinder the generalization of the data. In addition, it is suggested that more models should be tested in order to look for an association with a higher effect size.

It is necessary to further expand these findings with research within the same population and region, employing prospective cohorts or possible clinical trial developments. Such an approach will afford the inclusion of a more comprehensive dataset and enhance the control over variables. Our study was performed at a single medical center, limiting the generalizability of the results. Moreover, it is recommended that additional models be examined to identify associations with a greater effect size.

This study has highlighted a few important points worth noting. Our hospital unit is a reference center that provides care to the population without medical insurance. Thus, the incidence of GBS cases was higher than commonly reported, and the number of observations is one of the highest compared to other publications. To standardize the operationalization of the data, we excluded those with a high percentage of losses to avoid manipulating the analysis. The ultimate goal of this study is to develop a multivariate prognostic model that predicts which patients with GBS will require IMV. This will provide a standardized tool for managing these cases in our population.

In conclusion, the likelihood of requiring invasive mechanical ventilation for individuals with Guillain-Barré syndrome can be predicted by the presence of bulbar dysfunction, dysautonomia, and lower Medical Research Council (MRC) scores. This information can be used to guide care by identifying patients who may need closer monitoring, ICU admission, and IMV.

## Data availability statement

The raw data supporting the conclusions of this article will be made available by the authors, without undue reservation.

## Ethics statement

The studies involving humans were approved by Comité de ética e investigación del Hospital Civil de Guadalajara “Fray Antonio Alcalde.” The studies were conducted in accordance with the local legislation and institutional requirements. Written informed consent for participation was not required from the participants or the participants’ legal guardians/next of kin in accordance with the national legislation and institutional requirements.

## Author contributions

AR-M: Conceptualization, Investigation, Project administration, Writing – original draft, Writing – review & editing. JB-R: Conceptualization, Supervision, Writing – review & editing. FR-R: Data curation, Investigation, Writing – review & editing. JS-E: Data curation, Investigation, Writing – review & editing. LA-G: Data curation, Investigation, Writing – review & editing. KD-R: Writing – review & editing. GL-J: Investigation, Writing – review & editing. ES-R: Investigation, Writing – review & editing. CZ-R: Conceptualization, Data curation, Formal analysis, Methodology, Software, Supervision, Validation, Writing – review & editing.
