# Supplementary material for: Clinical predictors for mechanical ventilation assistance in Guillain-Barré syndrome
Source: Front Neurol. 2024 May 9;15:1385945. doi: 10.3389/fneur.2024.1385945 (PMC11111953; doi:10.3389/fneur.2024.1385945)
Supplement: Supplementary file 1 [file Table_1.DOCX]

**Supplementary material**

**Table 1**

| **Supplementary Table 1. Clinical characteristics of the patients regarding neurophysiological subtypes.** | | | | | | |
| --- | --- | --- | --- | --- | --- | --- |
| **Variable** | **AMAN** | **AMSAN** | **AIDP** | **ASAN** | **Indeterminado** | **P** |
| N (%) | 103 (46.4) | 43 (19.37) | 22 (9.91) | 3 (1.35) | 51 (22.97) | - |
| Median age (SD) | 39.16 (17.21) | 44.9 (16.82) | 35.45 (12.37) | 39 (5.29) | 38.29 (16.26) | 0.19 |
| Men - N (%) | 62 (45.93) | 34 (25.19) | 14 (10.37) | 0 (0) | 25 (18.52) | **0.007** |
| Women - N (%) | 41 (47.13) | 9 (10.34) | 8 (9.2) | 3 (3.45) | 26 (29.89) |  |
| **Clinical form - N (%)** | | | | | | |
| Classic form | 101 (47.42) | 42 (19.72) | 20 (9.39) | 2 (0.94) | 48 (22.54) | **0.043** |
| Miller-Fisher | 2 (25) | 1 (12.5) | 1 (12.5) | 1 (12.5) | 3 (37.5) |  |
| Facial diplegia | 0 (0) | 0 (0) | 1 (100) | 0 (0) | 0 (0) |  |
| **Precedent events - N (%)** | | | | | | |
| GI infection | 41 (51.9) | 11 (13.92) | 10 (12.66) | 1 (1.27) | 16 (20.25) | 0.37 |
| Respiratory tract infection | 19 (34.55) | 10 (18.18) | 7 (12.73) | 1 (1.82) | 18 (32.73) | 0.15 |
| Recent immunization | 6 (42.86) | 3 (21.43) | 1 (7.14) | 0 (0) | 4 (28.57) | 0.94 |
| **Weakness progression pattern - N (%)** | | | | | | |
| Ascendent | 68 (44.74) | 31 (20.39) | 15 (9.87) | 3 (1.97) | 35 (23.03) | 0.86 |
| Descendent | 35 (50) | 12 (17.14) | 7 (10) | 0 (0) | 16 (22.86) |  |
| **MRC weakness scale grade - μ (SD)** | | | | | | |
| Global weakness | 28.14 (14.85) | 31.56 (17.3) | 39.91 (14.6) | 44 (18.33) | 28.52 (16.37) | **0.01** |
| Upper limbs weakness | 15.02 (7.96) | 17.91 (10.15) | 21.18 (8.5) | 22.67 (8.08) | 16.04 (8.8) | **0.007** |
| Lower limbs weakness | 13.12 (8.2) | 13.65 (8.99) | 18.73 (8.41) | 21.33 (10.26) | 12.6 (9.03) | **0.04** |
| **Upper limbs myotatic reflexes - N (%)** | | | | | | |
| Arreflexia | 28 (46.67) | 12 (20) | 4 (6.67) | 1 (1.67) | 15 (25) | 0.56 |
| Hyporreflexia | 62 (47.33) | 26 (19.85) | 12 (9.16) | 1 (0.76) | 30 (22.9) |  |
| Normorreflexia | 10 (38.46) | 5 (19.23) | 6 (23.08) | 1 (3.85) | 4 (15.38) |  |
| Hyperreflexia | 3 (60) | 0 (0) | 0 (0) | 0 (0) | 2 (40) |  |
| **Lower limbs myotatic reflexes - N (%)** | | | | | | |
| Arreflexia | 39 (41.05) | 21 (22.11) | 4 (4.21) | 2 (2.11) | 29 (30.53) | **0.04** |
| Hyporreflexia | 54 (48.21) | 21 (28.75) | 15 (13.39) | 1 (0.89) | 21 (18.75) |  |
| Normorreflexia | 8 (66.67) | 1 (8.33) | 3 (25) | 0 (0) | 0 (0) |  |
| Hyperrreflexia | 2 (66.67) | 0 (0) | 0 (0) | 0 (0) | 1 (33.33) |  |
| **Cranial neuropathy N (%)** | | | | | | |
| Facial palsy | 7 (19.44) | 12 (33.33) | 9 (25) | 0 (0) | 8 (22.22) | **<0.001** |
| Bulbar dysfunction | 22 (33.85) | 16 (24.62) | 8 (12.31) | 0 (0) | 19 (29.23) | **0.098** |
| Oftalmoplexia | 8 (25.81) | 7 (22.58) | 4 (12.9) | 2 (6.459 | 10 (32.26) | **0.021** |
| Dysautonomias N (%) | 37 (45.68) | 13 (16.05) | 6 (7.41) | 1 (1.23) | 24 (29.63) | 0.39 |
| **Vital signs - μ (SD)** | | | | | | |
| SO2 | 94.69 (4.29) | 95 (4.02) | 95.63 (2.08) | 95.33 (2.51) | 95.05 (3.28) | 0.42 |
| RR | 18.59 (3.66) | 18.59 (3.92) | 18 (2.28) | 16 (2) | 18.92 (3.18) | 0.4 |
| HR | 84.42 (12.78) | 79.7 (17.06) | 85.9 (13.1) | 84 (4) | 84.33 (17.75) | 0.38 |
| **Hughes disability scale grade at admission- N (%)** | | | | | | |
| 1 | 5 (38.46) | 1 (7.69) | 4 (30.77) | 1 (7.69) | 2 (15.38) | **0.01** |
| 2 | 5 (38.46) | 4 (30.77) | 3 (23.08) | 0 (0) | 1 (7.69) |  |
| 3 | 17 (45.95) | 10 (27.03) | 4 (10.81) | 1 (2.70) | 5 (13.51) |  |
| 4 | 64 (52.46) | 19 (15.57) | 10 (8.2) | 1 (0.82) | 28 (22.95) |  |
| 5 | 12 (32.43) | 9 (24.32) | 1 (2.70) | 0 (0) | 15 (40.54) |  |
| **AMAN**= acute motor axonal neuropathy **AMSAN**= acute motor and sensory axonal neuropathy **ASAN**= acute sensory axonal neuropathy **AIDP**= acute inflammatory demyeliating polyneuropathy **GI**= gastrointestinal **MRC**= Medical Research Council **SO2**= saturación de oxígeno **RR**= respiratory rate **HR**= heart rate | | | | | | |

**Table 2**

| **Supplementary table 2. Biochemical variables of patients regarding neurophysiological subtypes** | | | | | | | |
| --- | --- | --- | --- | --- | --- | --- | --- |
| **Variable** | **Obs.*** | **AMAN** | **AMSAN** | **AIDP** | **ASAN** | **Indeterminate** | ***p** |
| N (%) | - | 103 (46.4) | 43 (19.37) | 22 (9.91) | 3 (1.35) | 51 (22.97) | - |
| **Biochemical - μ (SD)** | | | | | | | |
| Hemoglobin | 220 | 15.47 (1.81) | 15.8 (1.61) | 15.67 (2.2) | 12.1 (2.87) | 15.09 (2.41) | 0.11 |
| Platelets | 220 | 289.9 (92.12) | 257.52 (85.45) | 274 (51.41) | 287 (138.6) | 281.24 (95.9) | 0.22 |
| Leukocytes | 220 | 11.4 (5.26) | 10.56 (4.88) | 10.21 (3.55) | 11.16 (1.11) | 10.49 (4.1) | 0.53 |
| Lymphocytes | 220 | 2.47 (1.22) | 2.22 (1.04) | 2.29 (0.78) | 2.63 (1.11) | 2.13 (1.23) | 0.23 |
| SII | 220 | 1194.83 (1050.42) | 1133.92 (1212.9) | 1005.18 (760.26) | 898.18 (563.8) | 1281.03 (1034) | 0.68 |
| Glucose | 218 | 108.72 (45.81) | 105.1 (25.15) | 99.82 (24.31) | 101 (67.09) | 108.4 (45.7) | 0.7 |
| eGFR | 221 | 113.47 (24.6) | 109.88 (19.84) | 112.41 (18.56) | 106.67 (25) | 114.78 (24.54) | 0.54 |
| Na+ | 215 | 137.03 (4.15) | 136.05 (4.18) | 136.52 (2.36) | 136.67 (6.11) | 136.12 (5.43) | 0.43 |
| K+ | 215 | 4.02 (0.45) | 4.05 (0.47) | 3.88 (0.37) | 3.8 (0.36) | 3.94 (0.58) | 0.48 |
| Ca++ | 210 | 9.34 (0.73) | 9.26 (0.63) | 9.47 (0.64) | 8.4 (0.5) | 9.31 (0.71) | 0.1 |
| Mg ++ | 41 | 2.05 (0.32) | 2.18 (0.16) | 2.25 (0.16) | NA | 1.85 (0.32) | 0.058 |
| LDH | 179 | 175.99 (62.15) | 167.06 (66.7) | 186.37 (84.78) | 164.67 (42.03) | 203.86 (81.52) | 0.08 |
| AST | 190 | 47.53 (76.15) | 39.91 (25.9) | 40.5 (21.67) | 27.33 (7.09) | 43 (42.11) | 0.38 |
| ALT | 190 | 54.2 (109.86) | 36.1 (19.36) | 55.45 (57.26) | 35.67 (12.86) | 42.32 (36.5) | 0.42 |
| Album | 189 | 3.88 (0.61) | 3.87 (0.71) | 4.11 (0.57) | 3.67 (0.55) | 3.98 (0.51) | 0.46 |
| CPK | 111 | 190.8 (350.74) | 182.75 (144.41) | 228 (272.61) | 118 (60.81) | 312.45 (570.39) | 0.86 |
| **Complementary studies - N (%)** | | | | | | | |
| Albumin-cytologic dissociation | 167 | 56 (42.42) | 26 (19.70) | 13 (9.85) | 3 (2.27) | 34 (25.76) | 0.94 |
| **AMAN**= acute motor axonal neuropathy **AMSAN**= acute motor and sensory axonal neuropathy **ASAN**= acute sensory axonal neuropathy **AIDP**= acute inflammatory demyelinating polyneuropathy **SII** = systemic inflammatory index **eGFR**= estimated glomerular filtration rate **LDH**= lactic dehydrogeanse **ALT**= alanine aminotransferase **AST**= aspartate aminotranferase **CPK**= creatin phosphokinase  *****Considered observations frequency | | | | | | | |

**Table 3**

| **Supplementary Table 3. Clinical characteristics of the patients regarding neurophysiological subtypes.** | | | | | | |
| --- | --- | --- | --- | --- | --- | --- |
| **Variable** | **AMAN** | **AMSAN** | **AIDP** | **ASAN** | **Undetermined** | **P** |
| N (%) | 103 (46.4) | 43 (19.37) | 22  (9.91) | 3  (1.35) | 51  (22.97) | - |
| Median age (SD) | 39.16 (17.21) | 44.9 (16.82) | 35.45 (12.37) | 39  (5.29) | 38.29  (16.26) | 0.19 |
| Men - N (%) | 62 (45.93) | 34 (25.19) | 14 (10.37) | 0  (0) | 25  (18.52) | **0.007** |
| **Clinical form - N (%)** | | | | | | |
| Classic form | 101 (47.42) | 42 (19.72) | 20 (9.39) | 2  (0.94) | 48  (22.54) | **0.043** |
| Miller-Fisher | 2  (25) | 1  (12.5) | 1  (12.5) | 1  (12.5) | 3  (37.5) |  |
| Facial diplegia | 0  (0) | 0  (0) | 1  (100) | 0  (0) | 0  (0) |  |
| **Weakness progression pattern - N (%)** | | | | | | |
| Ascendent | 68 (44.74) | 31 (20.39) | 15  (9.87) | 3  (1.97) | 35  (23.03) | 0.86 |
| Descendent | 35  (50) | 12 (17.14) | 7  (10) | 0  (0) | 16  (22.86) |  |
| **MRC weakness scale grade - (SD)** | | | | | | |
| Global weakness | 28.14 (14.85) | 31.56 (17.3) | 39.91 (14.6) | 44 (18.33) | 28.52  (16.37) | **0.01** |
| Upper limbs weakness | 15.02 (7.96) | 17.91 (10.15) | 21.18 (8.5) | 22.67 (8.08) | 16.04  (8.8) | **0.007** |
| Lower limbs weakness | 13.12 (8.2) | 13.65 (8.99) | 18.73 (8.41) | 21.33 (10.26) | 12.6  (9.03) | **0.04** |
| **Cranial neuropathy N (%)** | | | | | | |
| Facial palsy | 7 (19.44) | 12 (33.33) | 9  (25) | 0  (0) | 8  (22.22) | **<0.001** |
| Bulbar dysfunction | 22 (33.85) | 16 (24.62) | 8 (12.31) | 0  (0) | 19  (29.23) | **0.098** |
| Ophthalmoplegia | 8 (25.81) | 7  (22.58) | 4  (12.9) | 2 (6.459 | 10  (32.26) | **0.021** |
| Dysautonomia  N (%) | 37 (45.68) | 13 (16.05) | 6  (7.41) | 1  (1.23) | 24  (29.63) | 0.39 |
| **Hughes disability scale grade at admission- N (%)** | | | | | | |
| 1 | 5 (38.46) | 1  (7.69) | 4 (30.77) | 1  (7.69) | 2  (15.38) | **0.01** |
| 2 | 5 (38.46) | 4  (30.77) | 3  (23.08) | 0  (0) | 1  (7.69) |  |
| 3 | 17 (45.95) | 10 (27.03) | 4 (10.81) | 1  (2.70) | 5  (13.51) |  |
| 4 | 64 (52.46) | 19 (15.57) | 10  (8.2) | 1  (0.82) | 28  (22.95) |  |
| 5 | 12 (32.43) | 9  (24.32) | 1  (2.70) | 0  (0) | 15  (40.54) |  |
| **AMAN**= acute motor axonal neuropathy, **AMSAN**= acute motor and sensory axonal neuropathy **ASAN**= acute sensory axonal neuropathy **AIDP**= acute inflammatory demyelinating polyneuropathy **GI**= gastrointestinal **MRC**= Medical Research Council **SO2**= saturación de oxígeno **RR**= respiratory rate, **HR**= heart rate | | | | | | |
